# Long‐term trends in the phylogenetic and functional diversity of Anatidae in South China coastal wetlands

**DOI:** 10.1002/eap.2344

**Published:** 2021-06-07

**Authors:** Xianli Che, Min Zhang, Xuebing Zhao, Qiang Zhang, Yanyan Zhao, Anders Pape Møller, Fasheng Zou

**Affiliations:** ^1^ Guangdong Key Laboratory of Animal Conservation and Resource Utilization Guangdong Public Laboratory of Wild Animal Conservation and Utilization Institute of Zoology, Guangdong Academy of Sciences 105 west xingang road Guangzhou Guangdong 510260 China; ^2^ Ecologie Systématique Evolution Université Paris‐Sud, CNRS AgroParisTech Université Paris‐Saclay Bâtiment 362 Paris Orsay F‐91405 France

**Keywords:** Anatidae, China, coastal wetland, functional diversity, long‐term change, phylogenetic diversity

## Abstract

Species loss has attracted much attention among scientists for more than half a century. However, we have little information on the trends in phylogenetic and functional changes behind the species loss although this information is always asynchronous and important for conservation and management. We measured community trends in Anatidae (ducks and geese) for the last 50 yr to quantify trends in phylogenetic and functional diversity patterns coinciding with taxonomic historical dynamics. We used one‐way ANOVAs to test if there was a significant historical trend in communities of Anatidae. We characterized taxonomic, phylogenetic, and functional diversity of communities. For taxonomic diversity, we used species richness (SR). For phylogenetic diversity, we calculated the standardized effect size of mean pairwise distances (ses.MPD) and the standard effect size of mean nearest taxon distances (ses.MNTD) in communities. For functional diversity, we calculated functional richness (FRic), functional evenness (FEve), functional divergence (FDiv), and the community‐level weighted means (CWM) of trait values for diet, foraging stratum, and body mass, separately. From the 1950s to 2010s, species richness declined without significant trends. The ses.MNTD of Anatidae communities showed no clear trends. However, ses.MPD of Anatidae communities declined dramatically during this period. For functional diversity, functional evenness of diet, foraging stratum, body mass, and functional dispersion of diet, foraging stratum did not increase or decline significantly. However, functional evenness of all traits, functional richness, and functional dispersion of body mass showed declined trends. The basic phylogenetic diversity and species body mass of Anatidae communities declined significantly because of a declining trend in the relative independent branch of geese. This makes it more challenging for implement community recovery in the future. More attention in conservation biology should consider taxonomic diversity and asynchrony in phylogenetic and functional diversity.

## Introduction

The global decline in animals, especially birds, has been a central topic in ecology for nearly haft a century (Rosenberg et al. 2019). In the 1970s, there were 529 bird species approaching 3 billion individuals in North America, however, over the last 48 yr, 303 of those species suffered declines with a net loss of approximately 29% of total abundance (Rosenberg et al. 2019). In Europe, a study using a 30‐yr data set of 144 bird species revealed that avian abundance declined while most of this decline was attributed to common species (Inger et al. 2015). Such declines are also present in Asia (Xu et al. 2017), Australia (Lindenmayer et al. 2018), and South Africa (Cooper et al. 2017). However, fewer studies focus on phylogenetic and functional diversity pattern trends that coincide with species population declines. Even though such patterns may be asynchronous with the taxonomically dynamic, it may provide more detailed information on the relationship among the species that declined the most. This raises questions about the kinds of ecosystem functions that these species provided? This information is important for slowing the loss of biodiversity and for the recovery of specific wildlife habitats in conservation (Tucker et al. 2017). Integrating phylogenetic methods and functional traits in ecology can improve our understanding of relationships among taxonomic, phylogenetic, and functional diversity and thereby offer new opportunities for conservation management (Devictor et al. 2010).

Phylogenies are increasingly incorporated into community ecology because phylogenetic diversity can reveal the full “tree of life” in evolutionary perspectives, making it easier to adopt development of gene sequence technology (Tucker et al. 2017). Changes in trait space can influence the provision of key ecosystem services, such as nutrient cycles, pest control, seed dispersal, pollination, and modification of the environment in ways that benefit other species (Wood et al. 2015). The relationships between phylogenetic and functional diversity among co‐occurring species are excellent representatives as proxies for community assembly processes (Ovaskainen et al. 2017). For example, clustering of phylogenetic diversity may indicate environmental filtering processes, some clades of the community being gradually excluded (Webb et al. 2002, Cosset and Edwards 2017). High functional dispersion can account for relative high abundances by shifting the position of the trait space centroid toward the most abundant species to highlight competition‐dominated processes (Laliberté and Legendre 2010).

Previous research on long‐term trends in plant phylogenetic and functional diversity showed that these could be separated into two realms: natural community colonization and human‐disturbed community restoration (Li et al. 2015). In natural communities, early colonizers were convergent toward species that were closely related and functionally similar, while later colonizers became divergent species that were less similar (Li et al. 2015). While in human‐disturbed communities, logging, for example, increased phylogenetic and functional dispersion in understory plants (Döbert et al. 2017). However, fire management of plant communities became increasingly restricted to subsets of more closely related species that were more phylogenetically clustered at tallgrass prairie remnants (Larkin et al. 2015). Thus, long‐term trends in phylogenetic and functional diversity reflected different colonization processes under different selection pressure. However, birds, as highly mobile living beings, may present different patterns compared to plants in long‐term trends (Che et al. 2019). Anatidae are among the most widespread and best known birds, being present on most major islands and continents (Dalby et al. 2014). They are important components of wetland biodiversity, providing important ecological functions. However, they are protected under international conventions due to legal quarry and thus of great societal and economic importance (Dalby et al. 2014). Historically, migratory Anatidae were an important protein source for prehistoric human hunter‐gatherers (MaMing et al. 2012) and recreational hunters in the United States kill more than 1 million ducks and geese during a hunting season (Raftovich et al. 2018). Despite their importance, no studies that quantify the phylogenetic and functional historical trends of Anatidae exist.

We used a long‐term data set to examine changes in the relationships among species diversity, phylogenetic diversity, and functional diversity of Anatidae from the 1950s to the 2010s. Based on our findings, we discuss (1) the potential value of phylogenetic and functional approaches as a complementary approach to taxonomic diversity, using information on monitoring, management, and restoration; and (2) the conservation of Anatidae based on these three biodiversity dimensions.

## Methods

### Anatidae community data sets

Anatidae species assemblages were obtained from monographs, literature, databases, and expert surveys. A data set from the 1950s was extracted from *Bird Distribution Catalogue in China I. Non‐Passeriformes* (Zheng 1955), with the data set from the 1960s extracted from Guan et al. (1963), the data set from the 1970s was extracted from *Bird Distribution List of China* (Zheng 1975), the data set from the 1980s was extracted from Deng et al. (1989), the data set from the 2000s was extracted from China Bird Report (*available online*), and a data set from the 2010s was extracted from our expert surveys.^4^ Each species assemblage for the 1950s, 1960s, 1980s, and 2010s included all species recorded used standardized methods as described (atlas, point count, territory mapping) implemented over at least three years. Data sets of the 1950s–1980s from books and literature were relatively independent investigations without overlap. Data sets from books (1950s and 1970s) included species but not abundance. Thus, we fitted models based on species richness data cautiously. Other studies have demonstrated that species‐based and abundance‐based phylogenetic and functional diversity showed a similar tendency (Li et al. 2015). Population trends deduced were based on data sets of the 1960s, 1980s, 2000s, and 2010s containing abundance data. Anatidae abundance of the 2000s was the largest single record of each species in the study region (Guangdong, China) during January 2000 to December 2010.

### Field work

The study wetlands were located in Guangdong Province, China (20°11′53.38″–23°37′14.05″ N, 109°40′15.94″–117°11′33.99″ E). The continental coastline of Guangdong is 3,368.1 km long, including 11 wetland types, with a total area of 8,150.98 km^2^, accounting for 46.49% of the wetland area in the whole province (Guo 2011).

From December 2014 to January 2018, we conducted point‐count waterbird surveys across Guangdong coastal wetlands in 101 field work days. We surveyed wetlands at 259 sites (distance between two sites >500 m), in 34 counties and 14 coastal cities that recorded waterbirds based on published papers, books, survey reports, and our fieldwork surveys since 2002. Those wetlands include intertidal mudflat, intertidal salt marsh, mangrove swamps, estuarine waters, estuarine deltas, lagoons, reservoirs, aquaculture ponds, paddy fields, and salt pans not more than 20 km from the coastal lines. Field surveys were completed on days with clear and calm weather, and the “point‐count” total counting method was employed by at least two experienced bird observers (X. Che and M. Zhang) with binoculars (8 × 42 WP Olympus; Olympus Corporation, Beijing, China) and a telescope (20–60× zoom ATM 80 Swarovski, Swarovski ATX25‐60X65, Absam, Austria) (Zou et al. 2006). The Anatidae taxonomy used is the same as in BirdTree (Jetz et al. 2012).

### Phylogenetic diversity data

We downloaded 2,000 phylogenetic trees of the complete Anatidae species pool in our study from BirdTree, the recently published phylogeny of the world's bird species, using the source of the Ericson All Species (Rubolini et al. 2015; data *available online*).^5^ We then calculated a maximum clade credibility tree with a 50% posterior probability limit by using the TreeAnnotator in the BEAST 2 software package (Bouckaert et al. 2014). Subsequent analyses of phylogenetic diversity were based on this annotated tree. Based on the phylogenetic tree, we calculated the species‐based standardized effect sizes of mean pairwise distance (ses.MPD) and the mean nearest taxon distance (ses.MNTD; Cadotte and Davies 2016). The MPD is an index calculating basic pairwise phylogenetic distances among co‐occurring species, representing a divergence at the family or genus level of the community (Cadotte and Davies 2016). The MNTD quantifies a terminal phylogenetic distance between nearest neighbors (sister species), describing the degree of species level (Cadotte and Davies 2016). To compare without directional bias associated with the variance of expected value decreasing with species richness increasing, we calculated a standardized effect size (ses) in null model analyses. A positive ses value (overdispersion) indicates a higher observation value than the average expected and a negative value (clustering) indicates a lower observation value than the average expected value.

### Functional diversity data

We quantified three functional traits for birds that are commonly used to define functional diversity within the bird communities, categorical variables diet, foraging stratum, and continuous variable body mass, which has been shown to be functionally important (Wilman et al. 2014). The diet category determined which type of food was eaten (e.g., invertebrates). Foraging stratum determined where foraging takes place (e.g., below the water surface). Body mass determined the physical attributes of the species (e.g., basal metabolic rate). We extracted those metrics from the Elton Traits database 1.0 (Wilman et al. 2014). Diet was classified into four categories and each category was ranked based on percentage used for each species: (1) invertebrates, (2) vertebrates and fish and carrion, (3) plant and seeds, and (4) omnivore. Foraging stratum was classified into three categories, and each category was also ranked based on the percentage used for each species: (1) below water surface, (2) water surface, and (3) ground. We calculated five complementary measures of functional diversity: functional richness (FRic), functional evenness (FEve), functional divergence (FDiv), functional dispersion (FDis), and the community‐level weighted means of trait values (CWM). FRic measures the unique trait value combinations in a community. Low FRic values indicate a low unique trait value (Villéger et al. 2008). FEve describes the distribution of species traits in a community across functional space. Low FEve values indicate that the community niches may be occupied and utilized inadequately, while high FEve values indicate community niches are occupied and utilized evenly (Villéger et al. 2008). FDiv relates to how functional trait distributed within the volume of trait space. The highest value of FDiv indicates that the most abundant species have extreme functional trait values (Villéger et al. 2008). FDis describes the mean distance of individual species to the centroid of all species in the community (Laliberté and Legendre 2010). For communities composed of only one species, FDis should be 0 (Anderson 2006). FDis has no upper limit. The CWM is an index of functional composition for an appointed trait in a community (Lavorel et al. 2008, Villéger et al. 2008).

### Data analyses

All analyses were performed in R 3.5.2 (R Core Team 2019). We used the sesmpd and sesmntd functions in the picante library to calculate the phylogenetic diversity metrics (Kembel et al. 2010). We used the dbFD function in the FD library to calculate the functional diversity metrics (Laliberté and Legendre 2010). We conducted one‐way ANOVAs in the multcomp library to tests significant trends (Hothorn et al. 2008).

## 1 Results

**Fig. 1:**
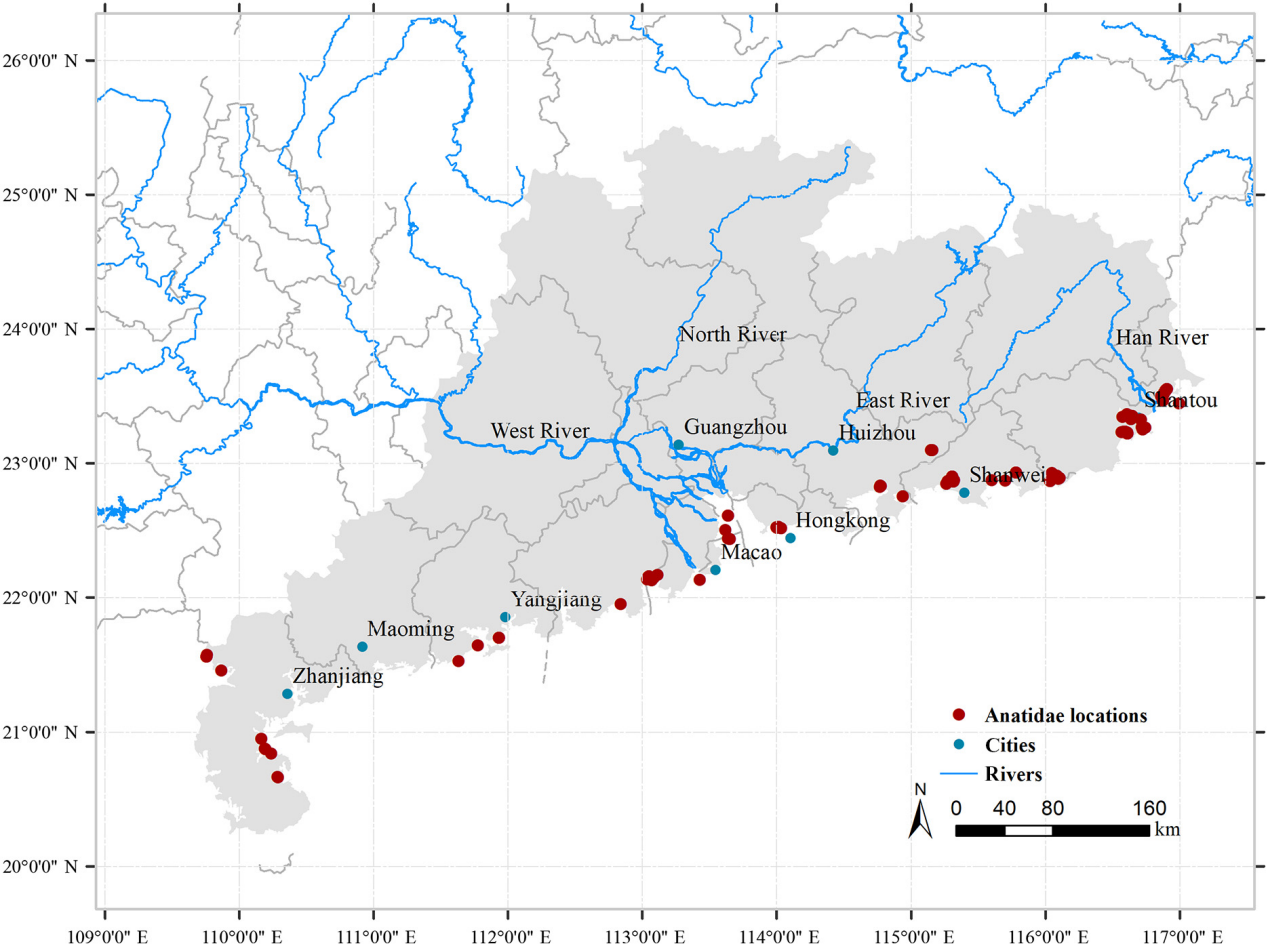
Map of localities where Anatidae were recorded in South China coastal wetlands during winter 2014–2018 created by ArcMap (V10.5). Solid dots indicated the location sites.

**Fig. 2:**
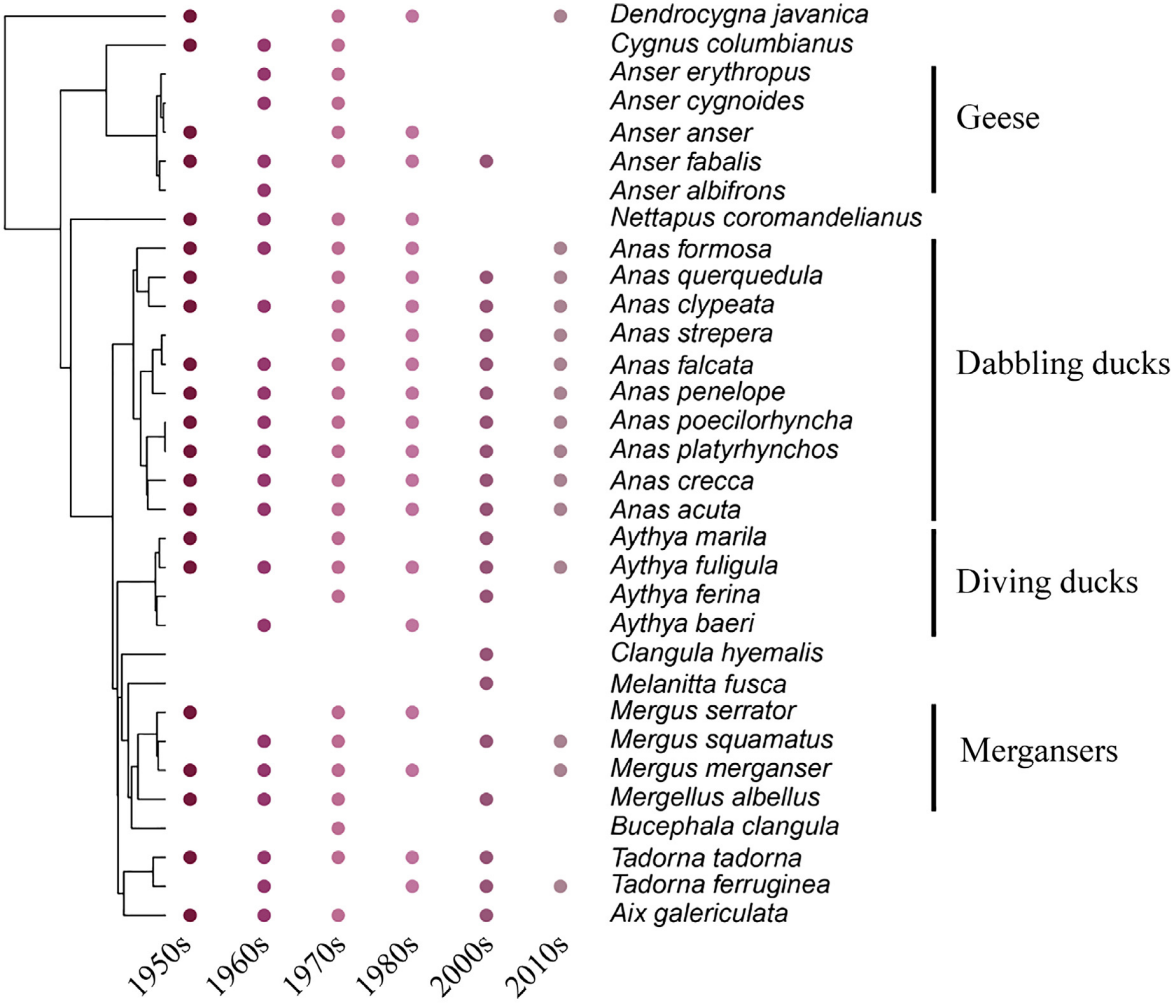
Species composition of each community and their relative phylogenetic relationship during the 1950s–2010s.

**Fig. 3:**
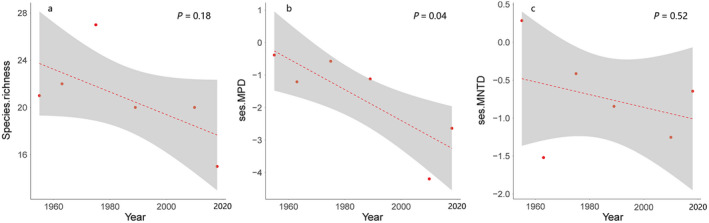
Historical trends of Anatidae communities in South China coastal wetlands from the 1950s to the 2010s. The temporal changes of species and phylogenetic diversity were measured as (a) species richness, (b) standardized effect sizes for mean pairwise phylogenetic distance (ses.MPD), and (c) mean nearest taxon phylogenetic distance (ses.MNTD), separately. The species richness (*P* = 0.18, a) and ses.MNTD (*P* = 0.52, c) decreased but without significant trends. The ses.MPD (*P* = 0.04, b) decreased significantly. We performed the analysis using occurrence‐based data. Each point shows the values of each age. The broken red line means the trends of each value. The dark gray area means the smooth fitting by using general linear models.

In total, during the wintering period of 2014‐2018, we recorded 15 duck species across Guangdong coastal wetlands (Fig. 1). The five dominant species were Northern Shoveler (*Anas clypeata*), Northern Pintail (*A*. *acuta*), Eurasian Wigeon (*A*. *penelope*), Green‐winged Teal (*A*. *crecca*), and Tufted Duck (*Aythya fuligula*), and those five species constituted more than 90% of total abundance. There were 21, 22, 27, 20, and 20 species recorded in the 1950s, 1960s, 1970s, 1980s, and 2000s, respectively (Fig. 2). Taxonomic diversity declined but without significant trends (*F*_1,4_ = 2.63, *P* = 0.18, Fig. 3a). Green‐winged Teal and Bean Goose (*Anser fabalis*) were the dominant species from 1950s to 1980s. However, Green‐winged Teal have declined more than 90% and Bean Geese have suffered local extinction since the 1950s.

**Fig. 4:**
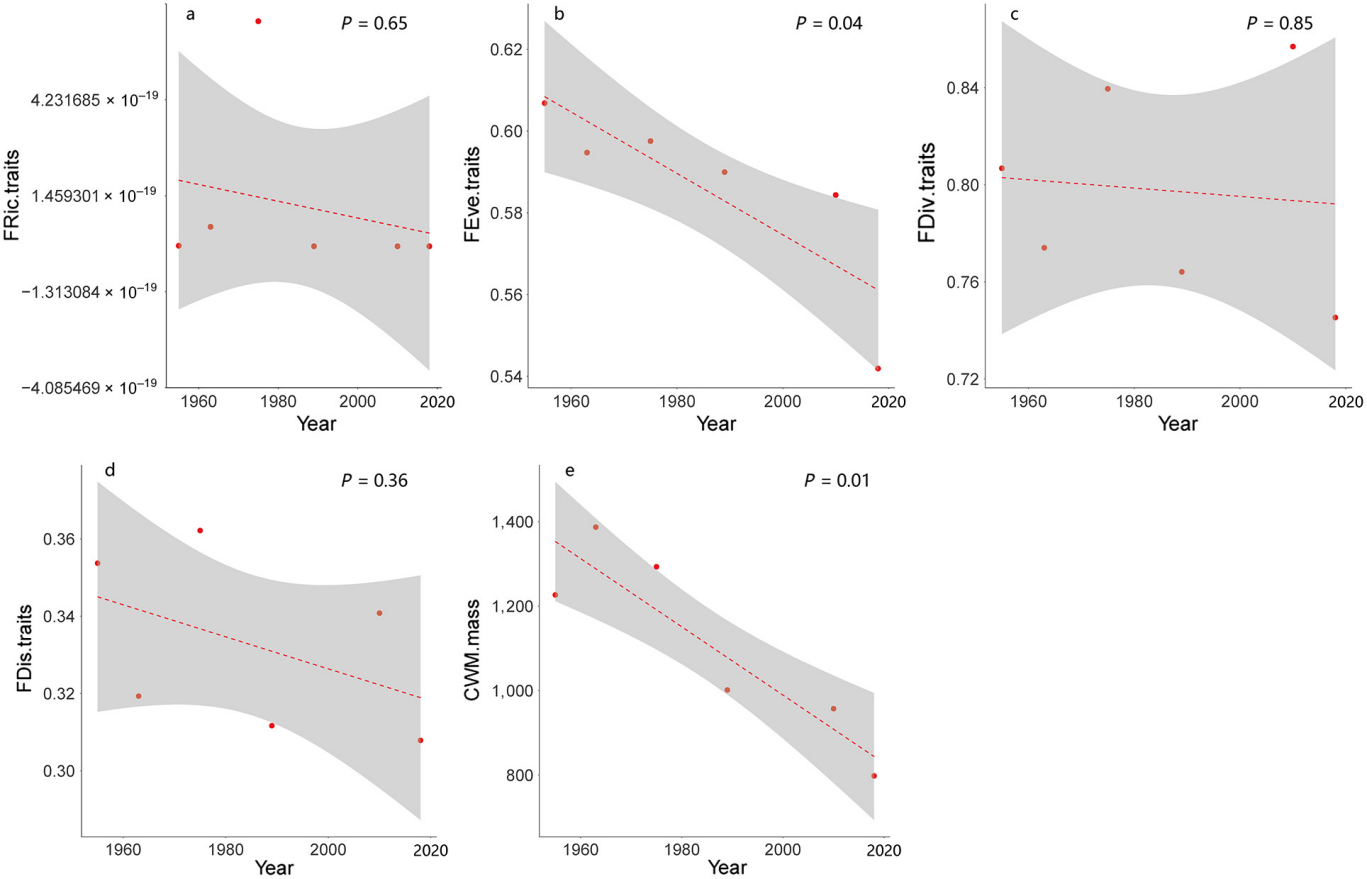
The temporal changes of functional diversity of total traits for (a) functional richness (*P* = 0.65), (b) functional evenness (*P* = 0.04), (c) functional divergence (*P* = 0.85), (d) functional dispersion (*P* = 0.36), and (e) community‐level weighted means of trait values of body mass (*P* = 0.01), separately. All the trends were declining. Figure components are as in Fig. 3.

**Fig. 5:**
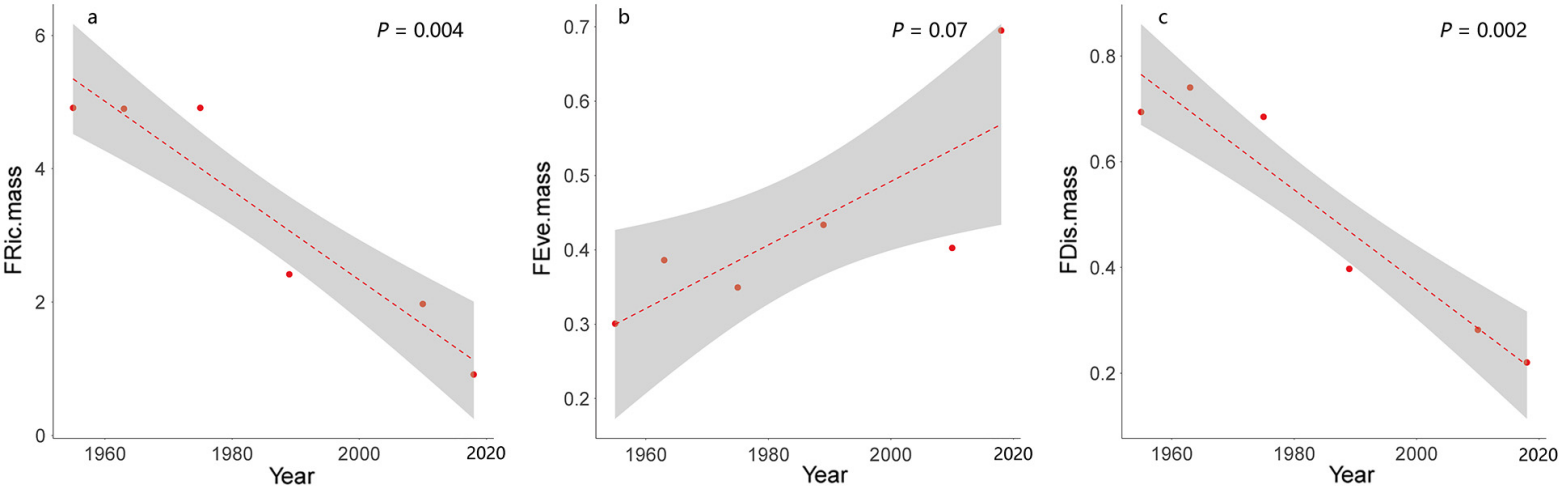
The temporal trends of body mass in Anatidae communities. (a) Functional richness (*P* = 0.004) declined with significant trends, (b) functional evenness (*P* = 0.07) increased but without significant trends, and (c) functional dispersion (*P* = 0.002) declined significantly, separately. Figure components are as in Fig. 3.

**Fig. 6:**
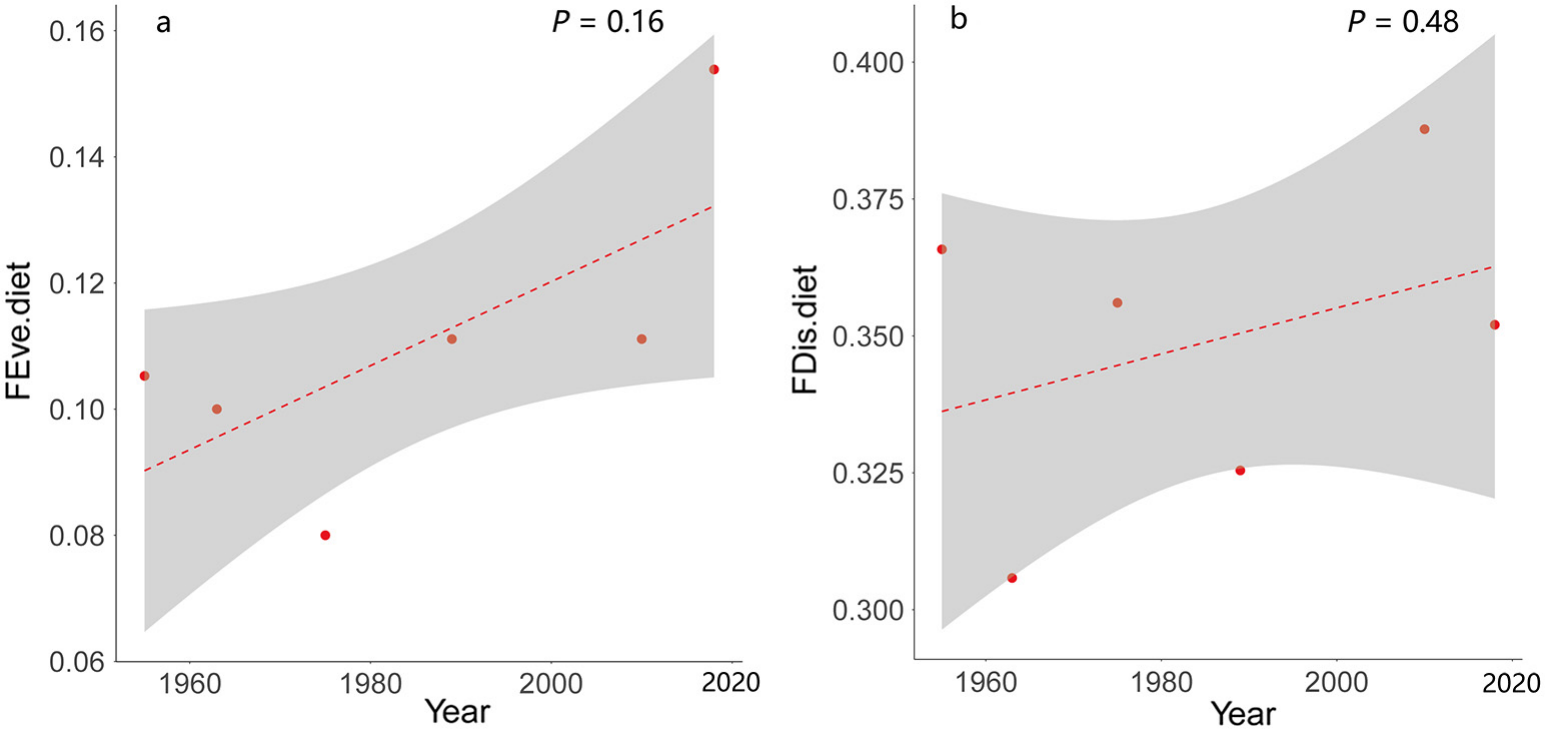
Historical trends of diet in Anatidae communities. Both (a) functional evenness (*P* = 0.16) and (b) functional dispersion (*P* = 0.48) increased without significantly. Figure components are as in Fig. 3.

**Fig. 7:**
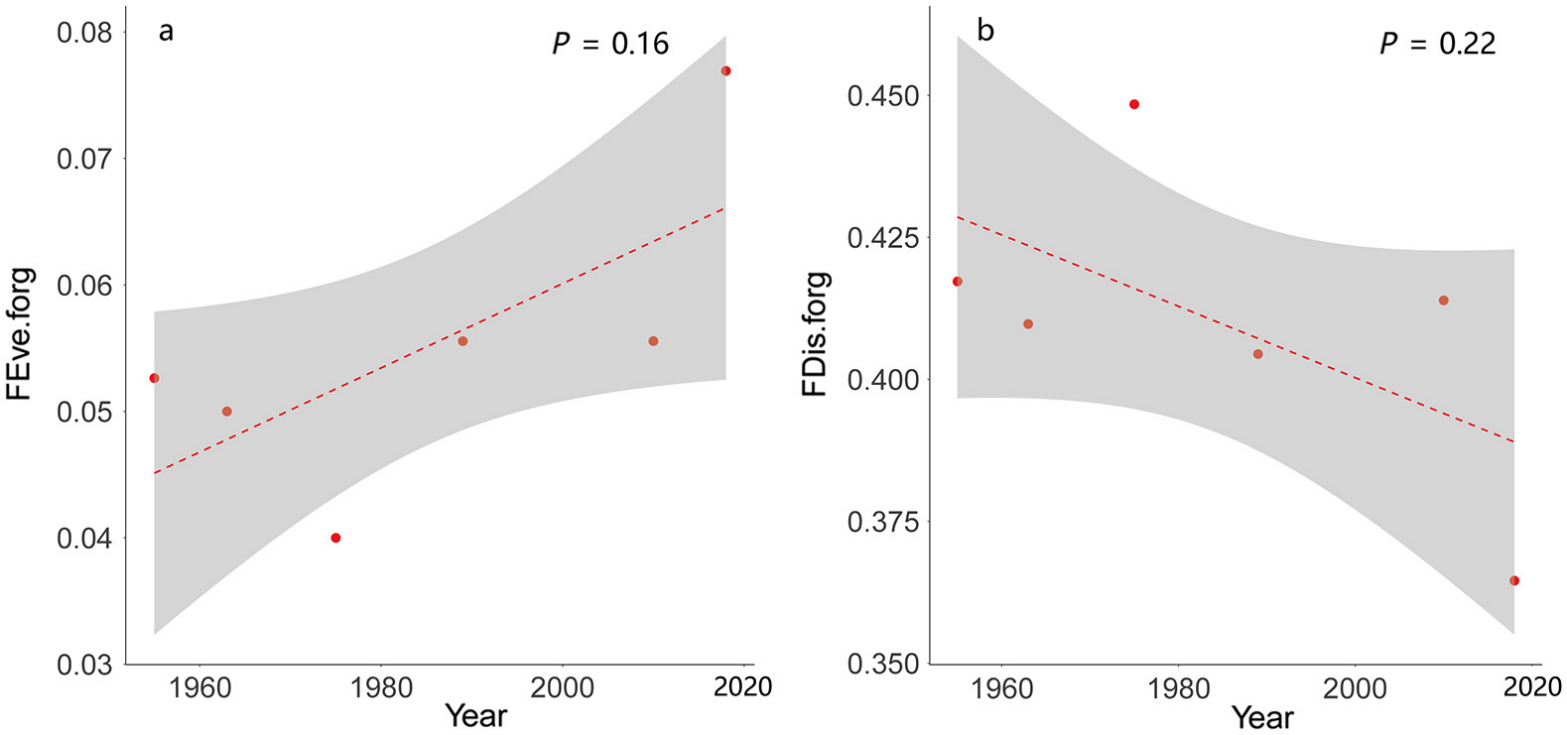
Historical trends of forage stratum in Anatidae communities. (a) The functional evenness (*P* = 0.16) increased but was not statistically significant; (b) functional dispersion (*P* = 0.22) decreased but was also not statistically significant. Figure components are as in Fig. 3.

For the phylogenetic perspective, the ses.MPD (*F*_1,4_ = 8.43, *P* = 0.04, Fig. 3b) declined significantly since the 1950s, even though the ses.MNTD (*F*_1,4_ = 0.50, *P* = 0.52, Fig. 3c) did not change significant. For all three traits totally, FRic (*F*_1,4_ = 0.23, *P* = 0.65, Fig. 4a), FDiv (*F*_1,4_ = 0.04, *P* = 0.85, Fig. 4c) and FDis (*F*_1,4_ = 1.07, *P* = 0.36, Fig. 4d) declined but with no signature. However, FEve (*F*_1,4_ = 9.12, *P* = 0.04, Fig. 4b) declined significantly. The CWM of mass (*F*_1,4_ = 18.07, *P* = 0.01, Fig. 4e) also declined significantly. For the mass separately, FEve (*F*_1,4_ = 6.25, *P* = 0.07, Fig. 5b) increased but not significantly. However, FRic (*F*_1,4_ = 36.39, *P* = 0.004, Fig. 5a) and FDis (*F*_1,4_ = 46.46, *P* = 0.002, Fig. 5c) declined significantly. The FEve (*F*_1,4_ = 3.75, *P* = 0.13, Fig. 6a) and FDis (*F*_1,4_ = 0.61, *P* = 0.48, Fig. 6b) of diet increased without significant trends. The FEve (*F*_1,4_ = 3.75, *P* = 0.16, Fig. 7a) of foraging stratum increased, however, the FDis (*F*_1,4_ = 2.14, *P* = 0.22, Fig. 7b) decreased; neither were statistically significant.

## 2 Discussion

Our research found that, in the South China coastal wetlands, the Anatidae community declined significantly at the genus level of phylogenetic diversity (ses.MPD). There were also significant declines in FEve of total traits, CWM, FRic, and FDis in the body mass of traits separately. Thus, we can conclude that even though the species richness trends did not decline significantly during the last 50 yr, a clade of Anatidae with large body mass declined significantly. This clade was represented by geese, such as the Bean Goose. More than 20,000 Bean Geese were recorded in the 1960s, about 10,000 in the 1980s, and none in the 2010s (Guan et al. 1963, Deng et al. 1989. Considering that phylogenetic diversity can reveal changes in species abundance patterns mediated by the influence of environmental conditions (Larkin et al. 2015), Anatidae in South China narrowed their ranges and declines in abundance were reflected by phylogenetic clusters. Phylogenetic diversity during plant succession based on only species richness data and abundance data also showed similar long‐term trends in field experiments (Li et al. 2015). The local extinction of the Bean Goose reflected by both phylogenetic and functional metrics but not by the taxonomic metric highlights the necessity of pluralistic approaches when measuring diversity. Some studies suggested that taxonomic, phylogenetic, and functional components of bird assemblages were similar among different habitats (Hanz et al. 2019). However, an enormous amount of research has revealed that, with the decline in species richness and functional diversity, phylogenetic diversity shows significantly different trends. Taxonomic, phylogenetic, and functional plant diversity of arable land to grassland showed different trends in different stages during a 270‐yr succession (Purschke et al. 2013). In the early to early‐mid stage, plant species richness increased but functional and phylogenetic diversity did not increase. In the mid‐late to late stage, functional diversity increased significantly but without species richness increasing. Seasonal patterns of waterbird taxonomic, phylogenetic, and functional diversity were synchronous in natural wetlands (Che et al. 2019), but asynchronous in man‐made subsidence wetlands (Li et al. 2019). Phylogenetic and functional clustering became more pronounced, maybe due to disturbance of environmental conditions. There is broad empirical support for disturbance, such as intensive monocultures, causing phylogenetic clustering (La Sorte et al. 2018). Close relatives often share similar environmental adaptations, resulting in niche sorting that characterized by phylogenetic clustering (Webb et al. 2002).

For the functional diversity, we found that only traits’ FEve showed significant decline. Considering that our Anatidae data set did not contain abundance, we concluded that the FEve (traits) decline was due to less regular functional distances among species. This irregularity is due to the partial disappearance of functional traits. The CWM values of diet (by plant and seeds) and foraging stratum (by water surface) did not change during the study periods while body mass declined significantly prompting us to separate the traits for further study. After splitting up the traits one by one for more details, we found that FRic and FDis of mass declined significantly while other indexes did not show significant trends. Thus, we concluded the traits’ FEve decline was due to mostly large‐sized geese and some medium‐sized ducks. Because the abundance data from the 1950s and 1970s is missing, we fitted the models with just species data. However, based on abundance data of 1960s, 1980s, 2000s, and 2010s, we can conclude that Anatidae population trends in South China coastal wetlands has declined dramatically, especially the geese. Those results also demonstrate that, when considering multiple functional traits in ecology, it is necessary to split traits one by one. Only by splitting traits can we discover which trait(s) was selected by the environmental press. This information is important in conservation practice.

Our findings illustrate that testing community species diversity relating to other aspects of diversity is important for making inferences about the trends or influences of community dynamics. However, obtaining trait data is required for such comparisons, limiting understanding of functional patterns without overcoming barriers of phylogeny (Larkin et al. 2015). Improvement of phylogenetic and functional ecology may help overcome this difficulty (Tucker et al. 2018). The development of tools and data‐sharing platforms has lowered barriers to integrating phylogenetic and functional analyses to traditional diversity indexes (Emerson and Gillespie 2008). There is a broad consensus that more work is necessary for screening the influence of phylogeny and function in applied contexts (Devictor et al. 2010, Winter et al. 2013). In our study, the phylogenetic and functional analysis provided insight into issues relevant for long temporal trends. For monitoring, phylogeny was a sensitive indicator of community change and species responses to environmental disturbance. Declining species were phylogenetically and functionally non‐random, suggesting that phylogenetic and functional diversity could inform species monitoring. However, there are limits to applying phylogenetic and functional information to management (Swenson 2013). Continued development of user‐friendly tools makes it possible to integrate phylogenetic and functional information into conservation and management (e.g., Jetz et al. 2012, Wilman et al. 2014). Besides, gaps in phylogenetic and functional knowledge remain under‐sampled for geographic regions and taxonomic groups, constraining our ability to use phylogenetic and functional data to guide conservation and management (Trindade‐Filho et al. 2012, Winter et al. 2013).

There were three important reasons why Anatidae declined in South China coastal wetlands. The first and most important is habitat loss. From 1992 to 2012, 42% or 760 km^2^ of the local wetlands were lost in the Pearl River Delta, the most important wetland region of South China (He et al. 2014). During this period, the Pearl River Delta experienced extremely rapid urban expansion: 33% of the land area was converted into urban use (4,169 km^2^; He et al. 2014). Wetlands have been lost at higher rates than any other ecosystem. China’s total urban land area increased with an annual average growth rate of 9% from 1992 to 2012, resulting in a total loss of wetland of more than 3,200 km^2^ in China, while the global annual average was only 3% over 1990–2000 (Angel et al. 2007). Previous global patterns of research found that urban assemblages contained fewer waterbird species and fewer water‐foraging species (La Sorte et al. 2018). Urban assemblages also contained a small proportion of species with large‐bodied and broadly distributed species (La Sorte et al. 2018). Compared to regional assemblages, urban assemblages maintained lower phylogenetic diversity, a loss of the relatively evolutionarily distant clades, and a reduction in evolutionarily closely related species (Morelli et al. 2016, Ibáñez‐Álamo et al. 2017).

The second factor contributing to the Anatidae decline is the lack of effective governance. Over the last 48 yr, waterfowl populations increased by 56% in North America, under the associated allocation of billions of dollars going toward wetland protection and restoration and adaptive hunting management, providing a model for proactive conservation (Rosenberg et al. 2019). How effectively the authorities exercise rules and enforcement mechanisms were the strongest predictor of global waterbird community abundance changes between 1990 and 2013 (Amano et al. 2018). Waterbird communities experienced increases in countries where governance was more effective but suffered significant declines in countries that had less effective governance (Amano et al. 2018). The effects of governance also interacted with those of protected area coverage (Amano et al. 2018). The coastal areas of East China had severely threatened, less protected, avian communities (Quan et al. 2018). Waterbird species in countries with rapidly expanding economies experienced greater declines (Ma et al. 2010). Governance was also the best predictor of population‐level abundance changes: less‐effective governance resulted in larger population‐level declines (Amano et al. 2018). Illegal hunting is the most conspicuous sign of ineffective governance with decreasing waterbird abundance (MaMing et al. 2012). Illegal trapping strongly contributed to the decline of the Yellow‐breasted Bunting (*Emberiza aureola*), one of the most abundant songbirds in China, with a population decline of 84–95% between 1980 and 2013 (Kamp et al. 2015). For waterbirds, the situation was more dire than for songbirds. Between 80,000 and 120,000 waterbirds belonging to 40 species were hunted every year, with geese, ducks, and swans accounting for the greatest abundance (MaMing et al. 2012). Poaching activities were very common during spring and autumn in China with ineffective hunting regulations. There is room for improvement in distributions of the currently protected areas, although most of the areas protect both taxonomic and phylogenetic diversity (Cui et al. 2014, Bai et al. 2015). Though the protected area systems of China comprised 428 national, 858 provincial, and 1,443 county nature reserves in 2014, avian communities at the coastal areas of East China were severely threatened, especially waterbirds (Quan et al. 2018). Indeed, 44% of internationally important sites, supporting more than 20,000 waterbirds or 1% of the population of an individual species, at coastal wetland sites were unprotected and none were well protected (Cui et al. 2014).

The third reason is climate change. Climatic change is among the most important abiotic factors affecting wildlife (Araújo and Rahbek 2006). Birds are shifting their ranges northeastward or eastward leading to population declines directly influenced by global warming (Both et al. 2006, Maclean et al. 2008, Both et al. 2009). Furthermore, the impact of global warming on waterbirds was more evident than the impact on terrestrial birds (Žalakevičius and Švažas 2005). In China, the inland Anatidae communities are being dominated by the larger species (geese and swans), and the coastal communities are being dominated by ducks with less body mass (Cao et al. 2008). However, distributions of 16 Anatidae were different from distributions before the 1970s (Cao et al. 2010). The Bean Goose has shifted its range northward, and abandoned the historical range in south China (Cao et al. 2010). Currently, most Bean Geese are found in the Yangtze floodplain or north of the Yangtze, the midlands of China (Cao et al. 2010). For the period 1906–2005, temperature anomalies showed a warming trend of 0.94°C in China (Wang et al. 2014). China’s surface air temperature continued to increase from the 1970s to the end of the 21st century (Wang et al. 2014). Species extinction risks will accelerate with future global temperatures, with 7.9% of species are predicted to become extinct from climate change (Urban 2015).

Our study illustrates that a comparative approach to investigate species, functional, and phylogenetic diversity and their historical trends is essential for detecting community patterns and dynamics. Most significantly, our findings suggest that phylogenetic and functional metrics were more sensitive to environmental filtering such as habitat loss or human disturbance. Hence, our findings indicate that phylogenetic and functional diversity were important complements, and not just a substitution, of species diversity. We conclude that further historical trend analysis in other ecosystems or classifications is needed to test for the generality of these results. For the south China Anatidae studied here, our findings emphasize that an asynchronous in taxonomic, functional, and phylogenetic diversity of bird assemblages should be considered in conservation.

Literature CitedAmano, T., T.Székely, B.Sandel, S.Nagy, T.Mundkur, T.Langendoen, D.Blanco, C. U.Soykan, and W. J.Sutherland. 2018. Successful conservation of global waterbird populations depends on effective governance. Nature553:199.2925829110.1038/nature25139Anderson, M. J.2006. Distance‐based tests for homogeneity of multivariate dispersions. Biometrics62:245–253.1654225210.1111/j.1541-0420.2005.00440.xAngel, S., J.Parent, D.Civco. 2007. Urban sprawl metrics: an analysis of global urban expansion using GIS. *In*Proceedings of ASPRS 2007 Annual Conference, Tampa, Florida. American Society for Photogrammetry and Remote Sensing ( ASPRS ), Bethesda, Maryland, USA.Araújo, M. B., and C.Rahbek. 2006. How does climate change affect biodiversity?Science313:1396–1397.1695999410.1126/science.1131758Bai, Q., et al. 2015. Identification of coastal wetlands of international importance for waterbirds: a review of China Coastal Waterbird Surveys 2005–2013. Avian Research
6:12.Both, C., S.Bouwhuis, C.Lessells, and M. E.Visser. 2006. Climate change and population declines in a long‐distance migratory bird. Nature441:81.1667296910.1038/nature04539Both, C., C. A.Van Turnhout, R. G.Bijlsma, H.Siepel, A. J.Van Strien, and R. P.Foppen. 2009. Avian population consequences of climate change are most severe for long‐distance migrants in seasonal habitats. Proceedings of the Royal Society B277:1259–1266.2001878410.1098/rspb.2009.1525PMC2842804Bouckaert, R., J.Heled, D.Kühnert, T.Vaughan, C.‐H.Wu, D.Xie, M. A.Suchard, A.Rambaut, and A. J.Drummond. 2014. BEAST 2: a software platform for Bayesian evolutionary analysis. PLoS Computational Biology10:e1003537.2472231910.1371/journal.pcbi.1003537PMC3985171Cadotte, M. W., and T. J.Davies. 2016. Phylogenies in ecology: a guide to concepts and methods. Princeton University Press, Princeton, New Jersey, USA.Cao, L., M.Barter, and G.Lei. 2008. New Anatidae population estimates for eastern China: implications for current flyway estimates. Biological Conservation141:2301–2309.Cao, L., Y.Zhang, M.Barter, and G.Lei. 2010. Anatidae in eastern China during the non‐breeding season: geographical distributions and protection status. Biological Conservation143:650–659.Che, X., D.Chen, M.Zhang, Q.Quan, A. P.Møller, and F.Zou. 2019. Seasonal dynamics of waterbird assembly mechanisms revealed by patterns in phylogenetic and functional diversity in a subtropical wetland. Biotropica51:421–431.Che, X., M.Zhang, X.Zhao, Q.Zhang, Y.Zhao, A.Møller, and F.Zou. 2021. Long‐term trends in the phylogenetic and functional diversity of Anatidae in South China coastal wetlands. Dryad, data set. 10.5061/dryad.xd2547dgbPMC845924233817885Cooper, T. J., A. M.Wannenburgh, and M. I.Cherry. 2017. Atlas data indicate forest dependent bird species declines in South Africa. Bird Conservation International27:337–354.Cosset, C. C., and D. P.Edwards. 2017. The effects of restoring logged tropical forests on avian phylogenetic and functional diversity. Ecological Applications27:1932–1945.2854399510.1002/eap.1578Cui, P., Y.Wu, H.Ding, J.Wu, M.Cao, L.Chen, B.Chen, X.Lu, and H.Xu. 2014. Status of wintering waterbirds at selected locations in China. Waterbirds37:402–410.Dalby, L., B. J.Mcgill, A. D.Fox, and J.Svenning. 2014. Seasonality drives global‐scale diversity patterns in waterfowl (Anseriformes) via temporal niche exploitation. Global Ecology and Biogeography23:550–562.Deng, J., G.Guan, B.Lu, and W.Chen. 1989. Bird diversity in Guangdong and Hainan, China (广东省及海南重要鸟类资源现况调查). Ecological Science (生态科学) 2:60–70.Devictor, V., D.Mouillot, C.Meynard, F.Jiguet, W.Thuiller, and N.Mouquet. 2010. Spatial mismatch and congruence between taxonomic, phylogenetic and functional diversity: the need for integrative conservation strategies in a changing world. Ecology Letters13:1030–1040.2054573610.1111/j.1461-0248.2010.01493.xDöbert, T. F., B. L.Webber, J. B.Sugau, K. J.Dickinson, and R. K.Didham. 2017. Logging increases the functional and phylogenetic dispersion of understorey plant communities in tropical lowland rain forest. Journal of Ecology105:1235–1245.Emerson, B. C., and R. G.Gillespie. 2008. Phylogenetic analysis of community assembly and structure over space and time. Trends in Ecology & Evolution23:619–630.1882367810.1016/j.tree.2008.07.005Guan, G., Y.Zhang, and T.Bei. 1963. A preliminary investigation on the wintering Anatidae in South China (我国南方越冬鸭类的初步调查). Chinese Journal of Zoology (动物学杂志)5:70–73.Guo, S.2011. Research on protection and management status of wetland resources in Guangdong and its countermeasures (广东湿地资源保护管理现状及其对策研究). Guangdong Forestry Science and Technology (广东林业科技)27:100–103.Hanz, D. M., K.Böhning‐Gaese, S. W.Ferger, S. A.Fritz, E. L.Neuschulz, M.Quitián, V.Santillán, T.Töpfer, and M.Schleuning. 2019. Functional and phylogenetic diversity of bird assemblages are filtered by different biotic factors on tropical mountains. Journal of Biogeography46:291–303.He, C., Z.Liu, J.Tian, and Q.Ma. 2014. Urban expansion dynamics and natural habitat loss in China: a multiscale landscape perspective. Global Change Biology20:2886–2902.2464399210.1111/gcb.12553Hothorn, T., F.Bretz, and P.Westfall. 2008. Simultaneous inference in general parametric models. Biometrical Journal50:346–363.1848136310.1002/bimj.200810425Ibáñez‐Álamo, J. D., E.Rubio, Y.Benedetti, and F.Morelli. 2017. Global loss of avian evolutionary uniqueness in urban areas. Global Change Biology23:2990–2998.2785999910.1111/gcb.13567Inger, R., R.Gregory, J. P.Duffy, I.Stott, P.Voříšek, and K. J.Gaston. 2015. Common European birds are declining rapidly while less abundant species' numbers are rising. Ecology Letters18:28–36.2536347210.1111/ele.12387Jetz, W., G.Thomas, J.Joy, K.Hartmann, and A.Mooers. 2012. The global diversity of birds in space and time. Nature491:444.2312385710.1038/nature11631Kamp, J., et al. 2015. Global population collapse in a superabundant migratory bird and illegal trapping in China. Conservation Biology
29:1684–1694.2605923310.1111/cobi.12537Kembel, S. W., P. D.Cowan, M. R.Helmus, W. K.Cornwell, H.Morlon, D. D.Ackerly, S. P.Blomberg, and C. O.Webb. 2010. Picante: R tools for integrating phylogenies and ecology. Bioinformatics26:1463–1464.2039528510.1093/bioinformatics/btq166La Sorte, F. A., et al. 2018. The phylogenetic and functional diversity of regional breeding bird assemblages is reduced and constricted through urbanization. Diversity and Distributions
24:928–938.Laliberté, E., and P.Legendre. 2010. A distance‐based framework for measuring functional diversity from multiple traits. Ecology91:299–305.2038021910.1890/08-2244.1Larkin, D. J., A. L.Hipp, J.Kattge, W.Prescott, R. K.Tonietto, S. K.Jacobi, and M. L.Bowles. 2015. Phylogenetic measures of plant communities show long‐term change and impacts of fire management in tallgrass prairie remnants. Journal of Applied Ecology52:1638–1648.Lavorel, S., K.Grigulis, S.McIntyre, N. S.Williams, D.Garden, J.Dorrough, S.Berman, F.Quétier, A.Thébault, and A.Bonis. 2008. Assessing functional diversity in the field–methodology matters!Functional Ecology22:134–147.Li, C., Y.Zhang, D.Zha, S.Yang, Z. Y. X.Huang, and W. F.de Boer. 2019. Assembly processes of waterbird communities across subsidence wetlands in China: a functional and phylogenetic approach. Diversity and Distributions25:1118–1129.Li, S., M. W.Cadotte, S. J.Meiners, Z.Hua, L.Jiang, and W.Shu. 2015. Species colonisation, not competitive exclusion, drives community overdispersion over long‐term succession. Ecology Letters18:964–973.2618964810.1111/ele.12476Lindenmayer, D. B., et al. 2018. Tests of predictions associated with temporal changes in Australian bird populations. Biological Conservation
222:212–221.Ma, Z., Y.Cai, B.Li, and J.Chen. 2010. Managing wetland habitats for waterbirds: an international perspective. Wetlands30:15–27.Maclean, I. M. D., et al. 2008. Climate change causes rapid changes in the distribution and site abundance of birds in winter. Global Change Biology
14:2489–2500.MaMing, R., T.Zhang, D.Blank, P.Ding, and X.Zhao. 2012. Geese and ducks killed by poison and analysis of poaching cases in China. Goose Bulletin15:2–11.Morelli, F., Y.Benedetti, J. D.Ibáñez‐Álamo, J.Jokimäki, R.Mänd, P.Tryjanowski, and A. P.Møller. 2016. Evidence of evolutionary homogenization of bird communities in urban environments across Europe. Global Ecology and Biogeography25:1284–1293.Ovaskainen, O., G.Tikhonov, A.Norberg, F.Guillaume Blanchet, L.Duan, D.Dunson, T.Roslin, and N.Abrego. 2017. How to make more out of community data? A conceptual framework and its implementation as models and software. Ecology Letters20:561–576.2831729610.1111/ele.12757Purschke, O., B. C.Schmid, M. T.Sykes, P.Poschlod, S. G.Michalski, W.Durka, I.Kühn, M.Winter, and H. C.Prentice. 2013. Contrasting changes in taxonomic, phylogenetic and functional diversity during a long‐term succession: insights into assembly processes. Journal of Ecology101:857–866.Quan, Q., X.Che, Y.Wu, Y.Wu, Q.Zhang, M.Zhang, and F.Zou. 2018. Effectiveness of protected areas for vertebrates based on taxonomic and phylogenetic diversity. Conservation Biology32:355–365.2870332510.1111/cobi.12986R Core Team
. 2019. R: a language and environment for statistical computing. R Foundation for Statistical Computing, Vienna, Austria.Raftovich, R. V., S. C.Chandler, and K. K.Fleming. 2018. Migratory bird hunting activity and harvest during the 2016–17 and 2017–18 hunting seasons. U.S. Fish and Wildlife Service, Laurel, Maryland, USA.Rosenberg, K. V., et al. 2019. Decline of the North American avifauna. Science
366:120–124.3160431310.1126/science.aaw1313Rubolini, D., A.Liker, L. Z.Garamszegi, A. P.Møller, and N.Saino. 2015. Using the BirdTree. org website to obtain robust phylogenies for avian comparative studies: a primer. Current Zoology61:959–965.3225653110.1093/czoolo/61.6.959PMC7098689Swenson, N. G.2013. The assembly of tropical tree communities–the advances and shortcomings of phylogenetic and functional trait analyses. Ecography36:264–276.Trindade‐Filho, J., F. L.Sobral, M. V.Cianciaruso, and R. D.Loyola. 2012. Using indicator groups to represent bird phylogenetic and functional diversity. Biological Conservation146:155–162.Tucker, C. M., et al. 2017. A guide to phylogenetic metrics for conservation, community ecology and macroecology. Biological Reviews
92:698–715.2678593210.1111/brv.12252PMC5096690Tucker, C. M., T. J.Davies, M. W.Cadotte, and W. D.Pearse. 2018. On the relationship between phylogenetic diversity and trait diversity. Ecology99:1473–1479.2978264410.1002/ecy.2349Urban, M. C.2015. Accelerating extinction risk from climate change. Science348:571–573.2593155910.1126/science.aaa4984Villéger, S., N. W.Mason, and D.Mouillot. 2008. New multidimensional functional diversity indices for a multifaceted framework in functional ecology. Ecology89:2290–2301.1872473910.1890/07-1206.1Wang, J., C.Xu, M.Hu, Q.Li, Z.Yan, P.Zhao, and P.Jones. 2014. A new estimate of the China temperature anomaly series and uncertainty assessment in 1900–2006. Journal of Geophysical Research: Atmospheres119:1–9.Webb, C. O., D. D.Ackerly, M. A.McPeek, and M. J.Donoghue. 2002. Phylogenies and community ecology. Annual Review of Ecology and Systematics33:475–505.Wilman, H., J.Belmaker, J.Simpson, C.de la Rosa, M. M.Rivadeneira, and W.Jetz. 2014. EltonTraits 1.0: Species‐level foraging attributes of the world's birds and mammals: Ecological Archives E095–178. Ecology95:2027.Winter, M., V.Devictor, and O.Schweiger. 2013. Phylogenetic diversity and nature conservation: where are we?Trends in Ecology & Evolution28:199–204.2321849910.1016/j.tree.2012.10.015Wood, S. A., D. S.Karp, F.DeClerck, C.Kremen, S.Naeem, and C. A.Palm. 2015. Functional traits in agriculture: agrobiodiversity and ecosystem services. Trends in Ecology & Evolution30:531–539.2619013710.1016/j.tree.2015.06.013Xu, Y., S.Lin, J.He, Y.Xin, L.Zhang, H.Jiang, and Y.Li. 2017. Tropical birds are declining in the Hainan Island of China. Biological Conservation210:9–18.Žalakevičius, M., and S.Švažas. 2005. Global climate change and its impact on wetlands and waterbird populations. Acta Zoologica Lituanica15:211–217.Zheng, Z.1955. Bird distribution catalogue in China I. Non‐Passeriformes (中国鸟类分布目录1·非雀形目). Science Press, Beijing, China.Zheng, Z.1975. Bird distribution list of China (中国鸟类分布名录). Science Press, Beijing, China.Zou, F., Q.Yang, T.Dahmer, J.Cai, and W.Zhang. 2006. Habitat use of waterbirds in coastal wetland on Leizhou Peninsula, China. Waterbirds29:459–464.

## Data Availability

Raw data (Che et al. 2021) are available from the Dryad Digital Repository: https://doi.org/10.5061/dryad.xd2547dgb

## Footnotes

^4^

http://www.birdreport.cn/

^5^

http://birdtree.org
